# Family quality of life in the context of Rett syndrome: insights from Brazilian families

**DOI:** 10.1007/s12687-026-00888-6

**Published:** 2026-04-28

**Authors:** Nicoly Stefani Sevalho Carlucci, Bianca Pereira Favilla, Beatriz Carvalho Nunes, Fernanda Teresa de Lima, Maria Isabel Melaragno, Rui Fernando Pilotto, Lucimar Retto da Silva de Avó, Carla Maria Ramos Germano, Débora Gusmão Melo

**Affiliations:** 1 Department of Medicine, Universidade Federal de São Carlos (UFSCar), São Paulo São Carlos, Brazil; 2 Genetics Division, Department of Morphology and Genetics, Escola Paulista de Medicina, Universidade Federal de São Paulo (UNIFESP), São Paulo São Paulo, Brazil; 3 Department of Gynecology, Escola Paulista de Medicina, Universidade Federal de São Paulo (UNIFESP), São Paulo, São Paulo Brazil; 4 Centro de Medicina Genômica, Hospital Israelita Albert Einstein, São Paulo, São Paulo Brazil; 5 Department of Genetics, Universidade Federal do Paraná (UFPR), Curitiba, Paraná Brazil; 6 Instituto Nacional de Genética Médica Populacional (INAGEMP), Porto Alegre, Brazil; 7 Escola Paulista de Medicina, Universidade Federal de São Paulo (UNIFESP), Rua Botucatu, 740. Edifício Leitão da Cunha. Disciplina de Genética. Vila Clementino, São Paulo, CEP 04023-900 SP Brazil

**Keywords:** Rett syndrome, Rare diseases, Quality of life, Family, Intellectual disability, Socioeconomic factors, Brazil

## Abstract

Rett syndrome (RTT) is a rare genetic neurodevelopmental disorder characterized by intellectual disability and severe functional impairments, placing a significant burden on affected families. This exploratory cross-sectional study evaluated family quality of life (FQoL) in Brazilian families of girls with RTT and examined its relationship with sociodemographic and clinical variables. Seventy Brazilian families participated in the study. Data were collected using sociodemographic and clinical questionnaires, the Pediatric Quality of Life Inventory, and the Beach Center Family Quality of Life Scale, which assesses five domains: family interaction, parenting, emotional well-being, physical and material well-being, and disability-related support. The average FQoL score was 3.68 ± 0.67, below the satisfaction threshold (4.0). Emotional well-being had the lowest score (3.16 ± 0.96), significantly lower than other domains (*p* < 0.001). Overall FQoL was associated with factors such as family income, access to welfare benefits and private health insurance, parental education and employment status, as well as individual characteristics of the person with RTT, including age, aggressiveness, and school type. Taken together, aggressiveness, attendance at a regular school, and a monthly income exceeding four official Brazilian minimum wages explained 29.8% of the variance in FQoL scores. The findings underscore the multifactorial nature of FQoL in the context of developmental disabilities. Improving FQoL for families of individuals with RTT requires integrated strategies that combine emotional support for caregivers, socioeconomic assistance, inclusive education, and medical and psychological care for individuals with RTT. These approaches have direct implications for public policies and service provision in low- and middle-income countries.

**Supplementary Information:**

The online version contains supplementary material available at 10.1007/s12687-026-00888-6.

## Introduction

Rett syndrome (RTT) is a rare genetic neurodevelopmental disorder that affects about 1 in 10,000 to 20,000 female live births. In addition, RTT is second most frequent genetic cause of intellectual disability in females, after Down syndrome (Asuncion and Ramani 2025). Phenotypically, RTT is characterized by a period of normal neurological and physical development with symptoms typically appearing between 6 and 18 months of age. The most common findings are partial or complete loss of intentional manual skills and speech, gait abnormalities, and the presence of stereotyped hand movements. Other symptoms may include growth delay, abnormal muscle tone, scoliosis or kyphosis, respiratory irregularities, bruxism, impaired sleep patterns, peripheral vasomotor disorders, and small and cold hands and feet (Asuncion and Ramani 2025; Kaur and Christodoulou 2019). Clinically, patients are categorized into typical and atypical forms. In about 95% of patients with typical RTT and 70% of those with atypical RTT, there is a pathogenic variant in the *MECP2* gene, located at Xq28 (Asuncion and Ramani 2025).

Given its broad physical and cognitive impact, RTT significantly affects not only the individuals diagnosed but also their families. There are two main studies that have assessed quality of life in individuals with RTT. In the first one, Bolbocean et al. (2022) used the Pediatric Quality of Life Inventory (PedsQL 4.0) to measure health-related quality of life (HRQoL) among children with syndromic autism spectrum disorder, which comprised Phelan-McDermid syndrome, SYNGAP1-related intellectual disability, and RTT. The study revealed that individuals with RTT had the lowest physical dimension score among the three disorders analyzed, while among people with RTT, the emotional domain had the highest ratings. Similarly, Rozensztrauch et al. (2021), also applying the PedsQL 4.0 in a sample of 23 individuals with RTT, found the lowest score in the physical dimension and the highest in the emotional dimension.

To better understand the impact of conditions such as RTT on family systems, the concept of Family Quality of Life (FQoL) has emerged as a valuable framework. FQoL refers to the collective well-being of all family members and the positive aspects of family life, particularly in the context of raising a child with a disability. Rather than focusing solely on the individual’s quality of life, FQoL highlights the dynamic interaction between individual and family-level needs, and how environmental and relational factors contribute to overall family functioning (Zuna et al. 2010; Boelsma et al. 2018). It encompasses multiple interrelated domains, including family interaction, parenting, emotional well-being, physical and material well-being, and disability-related support that reflect what families consider essential for a satisfying life (Brown and Brown 2014).

Over the past two decades, research on FQoL has evolved from deficit-based models of disability toward more holistic, family-centered approaches that recognize strengths, resilience, and the importance of meaningful goals once basic needs are met (Turnbull et al. 2000). In the field of disability studies, FQoL has become a critical tool for evaluating the real-life effects of services and policies, as it captures both the challenges and the resources available to families of individuals with developmental disabilities (Samuel et al. 2012). Understanding and measuring FQoL is particularly relevant in underrepresented contexts such as low- and middle-income countries, where structural inequalities may exacerbate the burden on families.

In practical terms, raising a child with intellectual and/or motor disabilities often brings profound changes to family dynamics and routines and is closely associated with caregiver burden. The literature describes a dynamic and bidirectional relationship between caregiver quality of life and FQoL, whereby parental stress, caregiving burden, and caregivers’ physical and emotional well-being are strong predictors of poorer outcomes across multiple family domains, including health, relationships, social support, and financial well-being (Barratt et al. 2025; Fernández-Ávalos et al. 2020; Jenaro et al. 2020). Conversely, interventions that provide emotional, social, and practical support to caregivers have been shown to improve overall FQoL (Boehm and Carter 2019; Hassanein et al. 2021). Families of children with disabilities also face additional demands related to specialized health knowledge, caregiving skills, access to material resources, and emotional support (Pelentsov et al. 2015; Staunton et al. 2023). Investigating FQoL is essential to identify support needs and inform strategies aimed at improving family functioning.

Specifically regarding RTT, some studies have shown that the condition negatively affects the quality of life of parents and caregivers (Laurvick et al. 2006; Sarajlija et al. 2013; Killian et al. 2016; Parisi et al. 2016; Corchón et al. 2018). However, there is a notable lack of research directly addressing FQoL in families of individuals with RTT. To our knowledge, only one study has explored this topic, reporting the lowest satisfaction in the emotional well-being domain (Bolbocean et al. 2022). Despite the growing interest in FQoL within disability research, its application to families affected by RTT remains limited, particularly in low- and middle-income countries, where access to specialized services and support is often restricted.

In this context, the present study aimed to examine trends in FQoL among Brazilian families of girls diagnosed with RTT. Specifically, we assessed family satisfaction across FQoL domains, evaluated aspects of HRQoL, and explored whether sociodemographic factors or individual characteristics of the child were associated with overall FQoL outcomes.

## Methods

### Study design and setting

This exploratory cross-sectional study was conducted with support from the Brazilian Association of Rett Syndrome (Associação Brasileira de Síndrome de Rett, ABRE-TE), a non-profit organization founded in 1990 to assist families, disseminate information, and advocate for public policies for individuals with RTT (https://abrete.org.br/). In 2021, about 400 families were registered in ABRE-TE.

This research was approved by the Human Research Ethics Committee of the Federal University of São Carlos (process CAAE 34586620.5.0000.5504), and participants provided informed consent.

### Sampling and procedure

A purposeful and convenience sampling strategy was used to recruit 70 families, all members of ABRE-TE. Inclusion criteria were (1) having a girl aged 2 to 25 years with RTT confirmed by genetic testing and (2) being accessible and willing to participate in the study.

Data were collected anonymously between November 1, 2020, and August 31, 2021, using an online form shared via Facebook and WhatsApp, due to the COVID-19 lockdown. The survey link was shared by ABRA-TE solely and directly to the participants. In 65 cases (92.9%), the respondent was the mother; in three cases (4.3%), it was the father; and in two cases (2.9%), the grandfather or grandmother.

### Data collection instruments

Data were collected using the “Family Sociodemographic Profile” and “Profile of the Person with RTT” questionnaires, developed by the researchers based on variables hypothesized to influence FQoL and HRQoL Supplementary Material 1.

Additional standardized instruments included the Pediatric Quality of Life Inventory (PedsQL 4.0; http://www.pedsql.org/) or the PedsQL 4.0 Generic Core Scales Young Adult Version and the Beach Center Family Quality of Life Scale (BCFQoLS; https://beachcenter.lsi.ku.edu/), all administered as parent proxy reports. The PedsQL assesses HRQoL in children, adolescents, and young adults aged 2 to 25 years, with scores ranging from 0 to 100; higher scores indicate better quality of life (Varni et al. 2007). Due to the constraints imposed by the COVID-19 pandemic, only the physical and emotional domains of the PedsQL were assessed, since the social and school domains would not have accurately reflected the participants’ reality at that time. The BCFQoLS evaluates family quality of life in families of individuals with disabilities using a 5-point Likert scale, with scores ≥ 4.0 indicating satisfaction (Hoffman et al. 2006). Both instruments have previously been validated in Portuguese (Jorge et al. 2015; Klatchoian et al. 2008).

### Participants

Participating families were from 16 of the 27 Brazilian federative units. Mothers had a mean age of 38.7 years (± 7.2), and fathers had a mean age of 41.6 years (± 9.1), both ranging from 22 to 63 years. Table 1 presents the families’ sociodemographic characteristics.

**Table 1:** Sociodemographic characteristics of families (*n* = 70)

Sociodemographic and family data	*n* (%)
Family composition	
2 people - only the child and her mother	6 (8.6)
3 or more people	64 (91.4)
Number of elderly people	
There were no elderly people in the house	60 (85.7)
1 elderly person	6 (8.6)
2 elderly people	4 (5.7)
Siblings	
Did not have siblings	29 (41.4)
Had at least one sibling	41 (58.6)
Monthly family income (R$)^†^	
Up to 2,090.00	26 (37.1)
Between 2,090.01 and 4,180.00	17 (24.3)
Between 4,180.01 and 10,450.00	18 (25.7)
Between 10,450.01 and 20,900.00	4 (5.7)
Above 20,900.01	5 (7.1)
Welfare benefits	
No	42 (60.0)
Yes	28 (40.0)
Private health insurance	
No	25 (35.7)
Yes	45 (64.3)
People covered by the health insurance (*n* = 45)	
All members of our family	29 (64.4)
Only children	5 (11.1)
Only the person with Rett syndrome	8 (17.8)
Others	3 (6.6)
Where the treatment of the person with Rett syndrome occurs	
Another city	19 (27.1)
The same city where the family lived	51 (72.9)
Religion	
Do not profess a religion	25 (35.7)
Profess a religion	45 (64.3)
Parental marital status	
Divorced or separated	23 (32.9)
Married or living together	47 (67.1)
Mother’s educational level	
Complete primary education or incomplete secondary education	3 (4.3)
Complete secondary education or vocational training course or incomplete higher education	24 (34.3)
Complete higher education or graduate studies	43 (61.4)
Mother’s employment status	
Did not work outside the home	28 (40.0)
Unemployed	6 (8.6)
Had a full- or part-time job	36 (51.4)
Father’s educational level	
Complete primary education or incomplete secondary education	13 (18.6)
Complete secondary education or vocational training course or incomplete higher education	28 (40.0)
Complete higher education or graduate studies	29 (41.4)
Father’s employment status	
Did not work outside the home	7 (10.0)
Unemployed	6 (8.6)
Had a full- or part-time job	57 (81.4)

†The Brazilian Real (R$) is the official currency of Brazil; US$1.00 = R$5.51 on August 5th, 2025

All girls with RTT had a confirmed pathogenic variant in the *MECP2* gene. Their mean age was 9.8 years (± 6.2). None were able to perform instrumental activities of daily living or manage money. Only one individual (1.4%) was able to perform basic self-care activities, such as bathing, using the toilet, dressing, or eating independently, although she required supervision. Table 2 presents some characteristics of the individuals with RTT.

**Table 2:** Characteristics of individuals with Rett syndrome (*n* = 70)

Personal and clinical characteristics of individuals with RS	*n* (%)
Age	
Between 2 and 4 years	17 (24.3)
Between 5 and 7 years	11 (15.7)
Between 8 and 12 years	23 (32.9)
Between 13 and 18 years	11 (15.7)
Between 19 and 25 years	8 (11.4)
Type of school	
She attended regular school	30 (42.9)
She attended a special school	17 (24.3)
She did not go to school	23 (32.9)
Epilepsy	
No. Never had seizures	18 (25.7)
Yes. Seizures are controlled with medications	33 (47.1)
Yes. Seizures are not controlled, even with medications	15 (21.4)
Yes. Seizures are controlled without needing to use medications	4 (5.7)
Spine alterations	
No	31 (44.3)
Yes	31 (44.3)
Unknown	8 (11.4)
Need for spine surgery (*n* = 31)	
No	14 (45.2)
Yes	4 (12.9)
Undetermined	13 (41.9)
Difficulty in socializing with peers	
No	54 (77.1)
Yes	16 (22.9)
Aggressiveness	
No	62 (88.6)
Yes	8 (11.4)
Difficulty of living together on a day-to-day basis	
No	54 (77.1)
Yes	16 (22.9)
Need to use medication for behavior	
No	42 (60.0)
Yes	28 (40.0)
Communication (*n* = 57)^†^	
No. It is very difficult to understand what she means, whether using words or non-verbal language	25 (43.9)
More or less. Usually, people in the family or those who live with her understand what she says, but strangers find it difficult to understand her	5 (8.8)
It is very difficult to understand what she says, but she communicates in a non-verbal way, and people in the family or around them understand what she means, but strangers do not understand	17 (29.8)
She communicates well non-verbally, and everyone understands what she means	10 (17.5)

†Excluding those under 3 years old

### Data analysis

Results were reported as percentages or as means ± standard deviations (SD), depending on the variable. The internal consistency of the BCFQoLS and PedsQL 4.0 was assessed using Cronbach’s alpha coefficient, with values ≥ 0.6 considered desirable (Taber 2018).

Normality of the overall FQoL score was verified with the Shapiro-Wilk test (W = 0.971; *p* = 0.105). Differences between BCFQoLS domain scores were evaluated using repeated-measures analysis of variance (ANOVA) with Greenhouse-Geisser correction and Bonferroni post hoc test.

Seven independent variables had more than two categories and were recoded into two groups for analysis as follows: Monthly family income was categorized as ≤ R$4,180.00, equivalent to four official Brazilian minimum wages at the time (0), and above that (1); maternal and paternal employment were coded as working outside the home (1) or not (0); maternal and paternal education were coded as having completed higher education (1) or not (0); school type was categorized as attending a regular school (1) or not (0); and epilepsy was categorized as present (1) or absent (0).

Binary associations between overall FQoL and independent variables were determined using the Pearson or Spearman correlations (continuous/ordinal variables) or t-tests with Cohen’s d estimates (dichotomous categorical variables). Multiple linear regression analyses were conducted in two stages: (1) variables with significant binary associations (*p* < 0.05) were entered simultaneously into the model; (2) based on the initial regression results, variables with the highest p-values were sequentially removed. At each step, the Akaike Information Criterion (AIC) and Schwarz’s Bayesian Information Criterion (BIC) were recorded. The final model was selected based on the lowest BIC value (Schwarz 1978). Multicollinearity among predictors was assessed using the variance inflation factor (VIF) and tolerance; VIF values < 10 and tolerance values > 0.10 were considered indicative of acceptable levels of collinearity (Hair et al. 2019).

Statistical analyses were performed using Jamovi 1.8.4.0 (https://www.jamovi.org), and *p* < 0.05 was considered statistically significant. The G*Power software (version 3.1.9.7) was used to calculate the statistical power of the multiple linear regression model (Faul et al. 2009).

## Results

Table 3 details the results of the BCFQoLS and the physical and emotional dimensions of the PedsQL 4.0. The mean overall FQoL score was 3.68 ± 0.67, and significant differences were found among the mean scores of the BCFQoLS domains (F(3.316, 228.782) = 19.779, *p* < 0.001, η²_p_=0.223). Individual BCFQoLS item responses are shown in Table S1 (Supplementary Information), with the number and percentage of individuals who were “very dissatisfied” (1) to “very satisfied” (5) for each of the 25 questions. Individual PedsQL 4.0 item responses for the physical and emotional domains are reported in Table S2 (Supplementary Information), including the number and percentage of individuals who responded “never” (100) to “almost always” (0) for each of the 13 questions in those domains. Mean BCFQoLS and PedsQL scores for each question are presented in Table S3 (Supplementary Information).

**Table 3:** Descriptive statistics for BCFQoLS and PedsQL 4.0 scores in families of children with Rett syndrome (*n* = 70)

		Mean (SD)	Median	Observed range	Cronbach’s alpha
Overall FQoL		3.68 (0.67)	3.74	2.16-5.0	0.882
BCFQoLS domains	Family interaction	3.80 (0.78)	4.0	1.33-5.0	0.876
	Parenting	3.74 (0.67)	3.83	2.0–5.0	0.832
	Emotional well-being	3.16 (0.96)	3.25	1.0–5.0	0.845
	Physical and material well-being	3.75 (0.86)	4.0	1.6-5.0	0.862
	Disability-related support	3.81 (0.85)	4.0	1.25-5.0	0.827
PedsQL 4.0 - physical dimension		38.57 (28.16)	31.25	0-96.88	0.861
PedsQL 4.0 - emotional dimension		63.93 (14.54)	65.0	20–95	0.549

Regarding the PedsQL results, the emotional domain presented significant binary associations with effective communication outside the family nucleus (t(55)=-2.526, *p* = 0.014, d=-0.880), difficulty in socializing with peers (t(68) = 2.384, *p* = 0.020, d = 0.678), aggressiveness (t(68) = 3.526, *p* < 0.001, d = 1.325), and difficulty of living together on a day-to-day basis (t(68) = 3.304, *p* = 0.002, d = 0.940). However, this was the only domain across both instruments to yield a Cronbach’s alpha below the acceptable threshold (α = 0.549). As for the physical domain, only the father’s educational level presented a significant binary association (U = 399, *p* = 0.020, rank biserial correlation = 0.329). Thus, PedsQL results were not considered for further analyses and the final model.

For the BCFQoLS, the Bonferroni test (Table S4, Supplementary Information**)** showed significant differences between the emotional well-being domain, which had the lowest score, and the following domains: family interaction (*p* < 0.001), parenting (*p* < 0.001), physical and material well-being (*p* < 0.001), and disability-related support (*p* < 0.001). The other four domains were not significantly different from each other.

Table 4 presents the relationship between FQoL and sociodemographic factors, family characteristics, and features of girls with RTT. Significant differences were identified in the mean FQoL scores according to family income (t(68) = 3.439, *p* < 0.001, d = 0.844), access to private health insurance (t(68)=-2.844, *p* = 0.006, d=-0.709), access to welfare benefits (t(68) = 3.640, *p* < 0.001, d = 0.888), mother’s educational level (t(68)=-3.765, *p* < 0.001, d=-0.925), mother’s employment status (t(68)=-2.984, *p* = 0.004, d=-0.714), father’s educational level (t(68)=-2.150, *p* = 0.035, d=-0.522), and father’s employment status (t(68)=-2.191, *p* = 0.032, d=-0.673). Regarding patient characteristics, age (Spearman’s ρ = -0.243, *p* = 0.042), aggressiveness (t(68) = 2.973, *p* = 0.004, d = 1.117), and type of school attended (t(68)=-2.906, *p* = 0.005, d=-0.702) were also significantly associated with differences in the mean FQoL scores.

**Table 4:** Associations between overall FQoL, family sociodemographic characteristics, and features of individuals with Rett syndrome (*n* = 70)

Variables	Mean Overall FQoL ± SD	Overall FQoL	
		Effect size (*r*/rho/Cohen’s d)	*p*-value
Sociodemographic and family variables			
Mother’s age^1^			
NA	NA	-0.142	0.239^2^
Father’s age^1^			
NA	NA	-0.081	0.507^3^
Family composition			
2 people - only the child and her mother (*n* = 6)	3.81 (0.75)	0.210	0.624^4^
3 or more people (*n* = 64)	3.66 (0.67)		
Number of elderly people^1^			
NA	NA	0.042	0.729^3^
Siblings			
None (*n* = 29)	3.82 (0.63)	0.365	0.137^4^
One or more (*n* = 41)	3.58 (0.69)		
Monthly family income^5^			
Up to 4,180^6^ (*n* = 43)	3.47 (0.69)	0.844	< 0.001^4,***^
Above 4,180 (*n* = 27)	4.00 (0.51)		
Private health insurance			
No (*n* = 25)	3.38 (0.62)	-0.709	0.006^4,**^
Yes (*n* = 45)	3.84 (0.65)		
Welfare benefits			
Do not receive welfare benefits (*n* = 42)	3.90 (0.66)	0.888	< 0.001^4,***^
Receive welfare benefits (*n* = 28)	3.35 (0.56)		
Religion			
Do not profess a religion (*n* = 25)	3.71 (0.76)	0.779	0.07^4^
Profess a religion (*n* = 45)	3.66 (0.63)		
Parental marital status			
Divorced or separated (*n* = 23)	3.46 (0.81)	-0.486	0.06^4^
Married or living together (*n* = 47)	3.78 (0.57)		
Mother’s educational level			
Incomplete higher education or lower educational levels (*n* = 27)	3.33 (0.59)	0.925	< 0.001^4,***^
Complete higher education or graduate studies (*n* = 43)	3.90 (0.63)		
Mother’s employment status			
Does not work outside the home (*n* = 34)	3.44 (0.68)	0.714	0.004^4,**^
Works outside the home, full-time or part-time (*n* = 36)	3.90 (0.59)		
Father’s educational level			
Incomplete higher education or lower educational levels (*n* = 41)	3.53 (0.69)	-0.522	0.035^4*^
Complete higher education or graduate studies (*n* = 29)	3.88 (0.60)		
Father’s employment status			
Does not work outside the home (*n* = 13)	3.32 (0.64)	0.673	0.032^4*^
Works outside the home, full-time or part-time (*n* = 57)	3.76 (0.66)		
Personal and clinical features of individuals with RTT			
Age^1^			
NA	NA	-0.243	0.042^3,*^
Type of school			
Did not attend a regular school (*n* = 40)	3.48 (0.66)	-0.702	0.005^4,**^
Attended a regular school (*n* = 30)	3.93 (0.61)		
Epilepsy			
Absent (*n* = 18)	3.77 (0.85)	0.193	0.484^4^
Present (*n* = 52)	3.64 (0.61)		
Spine alterations^7^			
Absent (*n* = 31)	3.85 (0.66)	0.458	0.076^4^
Present (*n* = 31)	3.55 (0.69)		
Need for spine surgery (*n* = 31)^8^			
Absent (*n* = 14)	3.76 (0.66)	-0.026	0.963^4^
Present (*n* = 4)	3.42 (0.67)		
Difficulty in socializing with peers			
Absent (*n* = 54)	3.76 (0.66)	0.516	0.704^4^
Present (*n* = 16)	3.41 (0.67)		
Aggressiveness			
Absent (*n* = 62)	3.76 (0.63)	1.117	0.004^4,**^
Present (*n* = 8)	3.05 (0.69)		
Difficulty of living together on a day-to-day basis			
Absent (*n* = 54)	3.74 (0.62)	0.380	0.186^4^
Present (*n* = 16)	3.48 (0.84)		
Necessity to use medication for behavioral issues			
Absent (*n* = 42)	3.76 (0.68)	0.311	0.207^4^
Present (= 28)	3.55 (0.66)		
Verbal communication (*n* = 57)^9^			
Did not have verbal communication (*n* = 52)	3.66 (0.65)	-0.058	0.902^4^
Had verbal communication (*n* = 5)	3.70 (0.35)		
Effective communication within the family nucleus (*n* = 57)^9^			
There was no effective communication (*n* = 25)	3.54 (0.64)	0.355	0.189^4^
There was effective communication (*n* = 32)	3.76 (0.62)		
Effective communication outside the family nucleus (*n* = 57)^9^			
There was no effective communication (*n* = 47)	3.65 (0.59)	-0.087	0.804
There was effective communication (*n* = 10)	3.71 (0.83)		

^1^Continuous variants; ^2^Person’s correlation; ^3^Spearman’s correlation; ^4^t-test; ^5^The Brazilian Real (R$) is the official currency of Brazil; US$1.00 = R$5.51 on August 5th, 2025; ^6^Correspondent to four Brazilian minimal wages at the time of the study; ^7^Unknown in eight patients; ^8^Undetermined in 13 patients; ^9^Excluding patients under 3 years old. *p < 0.05, **p < 0.01, ***p < 0.001

All significant variables identified in the bivariate tests (Table 4) were included in the multiple linear regression model presented in Table 5. After variable selection, the most parsimonious model - with the lowest BIC Supplementary Material 1- showed that “presence of aggressiveness,” “person with RTT attends regular school,” and “monthly family income above four official Brazilian minimum wages” were predictors of overall FQoL. The coefficient of determination for the final model indicated that it explained 29.8% of the variance in overall FQoL (R^2^ = 0.298, F(3, 66) = 9.336, *p* < 0.001). The model showed no multicollinearity issues, as VIF values did not exceed 2.0 and tolerance values were above 0.7. A post hoc power analysis revealed that a sample size of 70 was sufficient to detect a significant effect in a regression with 10 variables, as analyzed in this study, with a power of 0.99 and an alpha of 0.05. For the final model, the statistical power was also estimated at 0.99 with an alpha of 0.05, considering the same sample and three independent variables.

**Table 5:** Multiple linear regression of overall family quality of life (FQoL) based on family characteristics and features of individuals with Rett syndrome (*n* = 70)

Significant variables ANOVA *p* < 0.001 Durbin-Watson 1.891 (*p* = 0.640)			95% Confidence Interval					95% Confidence Interval		Collinearity Statistics		R^2^	F	AIC	BIC
Predictors	ß	SE	Lower	Upper	t	p	Stand ß	Lower	Upper	VIF	Tolerance				
Intercept	3.513	0.363	2.786	4.240	9.672	< 0.001						0.388	3.737	132.018	159.000
Monthly family income above four official Brazilian minimum wages	0.290	0.185	-0.081	0.661	1.565	0.123	0.431	-0.120	0.982	1.755	0.570				
Private health insurance	0.183	0.181	-0.180	0.546	1.009	0.317	0.272	-0.267	0.811	1.629	0.614				
Mother has completed higher education	0.129	0.222	-0.315	0.573	0.581	0.564	0.191	-0.468	0.850	2.511	0.398				
Father has completed higher education	-0.089	0.173	-0.435	0.257	-0.514	0.609	-0.132	-0.645	0.381	1.561	0.641				
Mother works outside the home	0.191	0.178	-0.165	0.548	1.074	0.287	0.284	-0.245	0.813	1.709	0.585				
Father works outside the home	0.221	0.193	-0.165	0.607	1.147	0.256	0.328	-0.244	0.901	1.211	0.826				
Person with RTT attends regular school	0.230	0.151	-0.072	0.532	1.525	0.133	0.341	-0.107	0.789	1.200	0.834				
Aggressiveness	-0.451	0.227	-0.906	0.004	-1.982	0.052	-0.669	-1.345	0.006	1.128	0.886				
Age of the person with RTT	-0.016	0.013	-0.043	0.010	-1.228	0.224	-0.148	-0.389	0.093	1.398	0.715				
Welfare benefits	0.037	0.222	-0.407	0.480	0.165	0.869	0.054	-0.604	0.713	2.543	0.393				
Intercept	3.413	0.113	3.188	3.638	30.314	< 0.001						0.298	9.336	127.604	138.846
Monthly family income above four official Brazilian minimum wages	0.466	0.143	0.180	0.751	3.261	0.002	0.691	0.268	1.114	1.014	0.986				
Person with RTT attends regular school	0.336	0.143	0.050	0.622	2.345	0.022	0.499	0.074	0.923	1.055	0.947				
Aggressiveness	-0.527	0.223	-0.972	-0.081	-2.360	0.021	-0.782	-1.443	-0.120	1.058	0.945				

## Discussion

Our sample primarily comprised families with three or more members who did not receive welfare benefits, had private health insurance, and lived in the same city where their child received medical care. Most girls with RTT attended regular schools, had seizures controlled with medication, and did not exhibit difficulties in social relationships or aggressiveness.

Although data collection occurred during the COVID-19 pandemic, a period that intensified caregiving challenges for families of children with disabilities, as professional support services were reduced or suspended (Neece et al. 2020; Mann et al. 2021; Navas et al. 2021), previous findings in the Italian population suggest that families of individuals with RTT may not have perceived greater parenting-related distress during this time. This may be because they already face elevated and persistent caregiving demands (Siracusano et al. 2021). Therefore, we hypothesize that the results presented in this study likely reflect the typical day-to-day experiences of these families rather than temporary effects related to the pandemic.

Our study assessed HRQoL using the PedsQL 4.0 instrument. Consistent with previous findings in North-American and Polish households of individuals with RTT (Bolbocean et al. 2022; Rozensztrauch et al. 2021), we found that the physical dimension scored lower on average than the emotional dimension. This pattern likely reflects the significant physical impairments characteristic of RTT. In contrast, the emotional dimension, while still impacted, may be comparatively less affected due to several factors. Parents and caregivers of Dutch individuals with profound intellectual and multiple disabilities often develop adaptive coping mechanisms and psychological resilience over time (Lahaije et al. 2024). Additionally, the emotional HRQoL dimension may be less directly influenced by the child’s physical limitations and more shaped by family support systems, access to resources, and psychological adjustment. The HRQoL scores observed in the present study may be attributed to sample characteristics, healthcare access, availability of support services, and cultural and socioeconomic factors, all of which may vary across study populations and settings.

Regarding FQoL in RTT, our findings also align with previous literature. Bolbocean et al. (2022) reported FQoL scores below the satisfaction threshold across all domains, with emotional well-being scoring lowest. This domain, composed of items addressing social support within and beyond the family, availability of external assistance, stress relief, and opportunities for individual autonomy among family members (Hoffman et al. 2006), also emerged as the most compromised in our study. A robust body of literature indicates that emotional well-being in families of individuals with intellectual disability is frequently compromised and encompasses anxiety, depression, social isolation, and anticipatory grief. Caregivers present a significantly increased risk of anxiety and depressive symptoms, particularly in contexts of greater disability severity, behavioral challenges, and limited social support (Scherer et al. 2019; Baker et al. 2021; Barratt et al. 2025). Social isolation and anticipatory grief have been consistently described in qualitative studies as sources of persistent emotional distress affecting caregivers’ mental health and family dynamics (Fernández-Ávalos et al. 2020; Rodrigues et al. 2019; Sardohan Yildirim et al. 2026). Factors such as resilience, perceived social support, and the meaning attributed to the caregiving experience may modulate the impact of these challenges (Jenaro et al. 2020; Hassanein et al. 2021; Sardohan Yildirim et al. 2026). In this sense, qualitative approaches could further elucidate the lived experiences underlying the emotional well-being domain and help contextualize quantitative findings. These results reinforce that emotional and psychological issues are central to the family experience in RTT and underscore the importance of addressing these dimensions in interventions aimed at improving FQoL. Although each FQoL domain provides valuable conceptual information, our analytical approach focused on overall FQoL to comprehensively capture this multidimensional construct and identify potential predictors.

Monthly family income above four official Brazilian minimum wages emerged as the strongest positive predictor of FQoL. This result aligns with previous studies demonstrating the significant impact of socioeconomic status on FQoL in Taiwanese, Canada-based and Catalan families with individuals with neurodevelopmental disorders (Chiu et al. 2020; Gardiner and Iarocci 2015; Giné et al. 2015). Caring for a person with a disability often involves additional direct costs (e.g., assistive equipment such as wheelchairs) and indirect costs (e.g., loss of income due to reduced employment by the primary caregiver), which can substantially affect household finances (Shahat and Greco 2021). North-American families of individuals with intellectual disabilities are more vulnerable to falling into poverty and less likely to overcome it (Rothwell et al. 2019). In Brazil, welfare benefits are only granted to very low-income families, which may explain why families that receive such benefits had lower FQoL in our t-test analysis. A study with Canadian families of children with autism spectrum disorder showed that higher family income facilitates access to private health insurance and services (Fong et al. 2020), which is also associated with higher parental education levels, a factor linked to improved FQoL among Chinese and Catalan households with individuals with intellectual disabilities (Giné et al. 2015; Hu et al. 2012). Higher educational attainment increases the likelihood of securing better-paying jobs (Rozensztrauch et al. 2021) and may also enhance access to relevant social services, support networks, and understanding of the child’s condition and needs for Taiwanese caregivers of individuals with profound intellectual and multiple disabilities (Chou et al. 2011). However, the relationship between education and FQoL is complex. Schnitzer et al. (2017) found that, in a German sample, caregivers with higher education levels may also experience greater psychological burden, potentially due to heightened awareness of lost autonomy and increased expectations. Further studies are needed to better understand the relationship between parental education and FQoL in RTT in Brazil.

Inclusive schooling also emerged as a significant positive predictor of FQoL. Despite national policies promoting inclusive education in Brazil, many families still face challenges enrolling their children in mainstream schools (Goés 2012). Furthermore, the quality of inclusion is often compromised by insufficient teacher training and limited access to appropriate educational resources (Cenci et al. 2020). These barriers may explain why a considerable portion of our sample attended special schools or did not attend school at all. In a North American sample, most children with RTT were enrolled in special classrooms or separate schools; however, families whose daughters attended more inclusive environments reported greater satisfaction with educational services (Larriba-Quest et al. 2020). This highlights the potential benefits of inclusive education for families of children with RTT. However, this association might not be generalizable beyond the studies’ target populations, as national education systems can vary. Although RTT is a neurodevelopmental disorder, individuals retain the potential to acquire new skills when appropriately stimulated, even into adulthood (Elefant and Wigram 2005). Since most individuals with RTT are non-verbal, educational professionals must be trained to implement assistive technologies, such as augmentative and alternative communication systems, and to adapt learning materials to meet their needs (Larriba-Quest et al. 2020; Vessoyan et al. 2018). Music therapy may also support learning by enhancing communication and interaction (Elefant and Wigram 2005).

We identified a modest negative association between the age of the individual with RTT and FQoL, suggesting that lifespan-related challenges may increasingly affect families over time. Studies in families of individuals with intellectual disability indicate that caregiver burden often persists and may intensify during adolescence and adulthood, reflecting cumulative caregiving demands, financial strain, reduced social support, uncertainty about the future, and sustained emotional burden (Barratt et al. 2025; Fernández-Ávalos et al. 2020; Jenaro et al. 2020). In RTT, this pattern may be related to the condition’s complex clinical profile, sustained high support needs, and growing concerns regarding long-term care. Evidence suggests that the negative impact on caregivers’ physical and emotional well-being intensifies as daughters with RTT age, particularly during adolescence and early adulthood, in association with increased caregiving demands, progression of comorbidities (e.g., sleep disturbances, behavioral problems, and feeding difficulties), longer duration of care, limited social support, and future-related concerns, contributing to increased parental stress and declines in family quality of life over time (Mori et al. 2019; Larsen et al. 2024; Pari et al. 2020).

The presence of aggressiveness in girls with RTT emerged as the strongest negative predictor of FQoL. While previous studies in Australian, Serbian, and Italian samples have associated clinical features such as motor impairments, feeding difficulties, seizures, sleep disturbances, and behavioral challenges with reduced quality of life for parents and caregivers, as well as changes in family dynamics (Laurvick et al. 2006; Sarajlija et al. 2013; Parisi et al. 2016), our findings emphasize the distinct and significant impact of aggressive behaviors. Corchón et al. (2018) reported that the mental health and well-being of parents are significantly influenced by RTT-related clinical features, with seizures and other comorbidities identified as major contributors to reduced FQoL. However, aggressiveness was not specifically addressed in their analysis. Our findings suggest that managing aggressive behavior in individuals with RTT should be prioritized in interventions aimed at improving family well-being. As we did not use a systematic instrument to evaluate behavior (including aggressiveness), our inferences remain limited. Nonetheless, this result highlights that studies addressing behavior and FQoL in individuals with RTT may further characterize this association. Further empirical research is warranted to explore the mechanisms underlying this association and to identify effective strategies for supporting families coping with behavioral challenges in RTT.

Taken together, our results point to three key areas for targeted intervention: financial support, inclusive education, and behavioral management. Families with higher income levels and those whose children attend regular schools report markedly better FQoL, while families dealing with aggressive behaviors report substantially lower FQoL. These findings underscore the need for integrated, interdisciplinary strategies to support family well-being in the context of developmental disabilities. They also suggest several potential avenues for practical application. Socioeconomic policies that enhance household income may contribute meaningfully to improved FQoL. Expanding access to inclusive education for girls with RTT may also yield broader benefits for the family system. Moreover, interventions focused on managing aggressive behavior could yield considerable improvements in FQoL, reinforcing the importance of behavior-focused support in care planning.

This study has some limitations. First, the cross-sectional design precludes causal inference. Thus, the absence of a comparison group without RTT or presenting another condition other than RTT limits the extrapolation of our findings beyond the RTT context. As a result, the extent to which the patterns in FQoL in RTT differ from those in other populations cannot be determined. Second, the use of a convenience sample restricted to those with internet access may limit generalizability due to potential selection bias. This may have limited the diversity within the sample, with a predominance of parents with higher education and private insurance, and restricted representation of more vulnerable families. Third, we relied on self-reported data collected via non-validated sociodemographic and clinical questionnaires, and did not use RTT specific severity scales (Raspa et al. 2020; Singh et al. 2022), which might have limited comparisons with previous studies. Lastly, data were primarily obtained from mothers, potentially limiting the representation of perspectives from other family members. Future research should incorporate a broader range of family perspectives and longitudinal designs to strengthen evidence on FQoL.

Despite these limitations, this study makes a novel contribution by being the first to assess FQoL in Brazilian families of girls with RTT, using a validated international scale. The sample included families from diverse socioeconomic backgrounds across various regions of Brazil, thereby strengthening the relevance of our findings. These results offer actionable insights for policy and practice, particularly in low- and middle-income settings where resources for families affected by developmental disabilities are often limited.

## Conclusions

This study demonstrates that FQoL among Brazilian families of girls with RTT is below the satisfaction threshold, with emotional well-being emerging as the most compromised domain. These findings underscore the complex and multifactorial nature of FQoL in the context of developmental disabilities, where emotional, educational, behavioral, and socioeconomic dimensions intersect.

To effectively support these families, integrated public policies are needed to promote emotional and psychological support for caregivers, improve access to health and social services, and expand welfare benefits to mitigate the financial burden often associated with caregiving. Additionally, advancing inclusive education policies and ensuring that schools are adequately equipped to support students with RTT are essential components of a comprehensive response.

Importantly, our results identify aggressiveness as a significant negative predictor of FQoL, underscoring the need for targeted behavioral interventions. Efforts to manage and reduce aggressive behaviors in individuals with RTT may represent one of the most impactful strategies for improving overall family well-being.

Ultimately, these results provide evidence to inform interdisciplinary strategies and policy development aimed at improving the daily lives of families affected by developmental disabilities, particularly in low- and middle-income countries. Our findings reinforce the need for coordinated efforts across health, education, and social protection sectors to enhance FQoL and promote meaningful inclusion for individuals with RTT and their families.

## Supplementary Information

Below is the link to the electronic supplementary material.

Supplementary Material 1 (DOCX 53.4 KB)

## Data Availability

All relevant data are within the paper and its supplemental files. A data management plan for this research is available at 10.48321/D1930S. Should anything else be needed, the corresponding author can be directly contacted at dgmelo@unifesp.br.
