# Supplementary material for: Family quality of life in the context of Rett syndrome: insights from Brazilian families
Source: J Community Genet. 2026 Apr 28;17(3):53. doi: 10.1007/s12687-026-00888-6 (PMC13121640; doi:10.1007/s12687-026-00888-6)
Supplement: Supplementary file 1 — Supplementary Material 1 (DOCX 53.4 KB) [file 12687_2026_888_MOESM1_ESM.docx]

**SUPPLEMENTARY INFORMATION**

**Family quality of life in the context of Rett syndrome: Insights from Brazilian families**

**Authors and affiliations**

Nicoly Stefani Sevalho Carlucci^1^^[[1]](#footnote-1)^

Bianca Pereira Favilla^2*^

Beatriz Carvalho Nunes^2*^

Fernanda Teresa de Lima^3,4*^

Maria Isabel Melaragno^2^

Rui Fernando Pilotto^5^

Lucimar Retto da Silva de Avó^1^

Carla Maria Ramos Germano^1^

Débora Gusmão Melo^2,6^

1 - Department of Medicine, Universidade Federal de São Carlos (UFSCar), São Carlos, São Paulo, Brazil.

2 - Genetics Division, Department of Morphology and Genetics, Escola Paulista de Medicina, Universidade Federal de São Paulo (UNIFESP), São Paulo, Brazil.

3 - Department of Gynecology, Escola Paulista de Medicina, Universidade Federal de São Paulo (UNIFESP), São Paulo, SP, Brazil.

4 - Centro de Medicina Genômica, Hospital Israelita Albert Einstein, São Paulo, São Paulo, Brazil.

5 - Department of Genetics, Universidade Federal do Paraná (UFPR), Curitiba, Paraná, Brazil.

6 - Instituto Nacional de Genética Médica Populacional (INAGEMP), Brazil.

**Correspondence to**

Débora Gusmão Melo

Universidade Federal de São Paulo (UNIFESP)

Escola Paulista de Medicina, Departamento de Morfologia e Genética

Rua Botucatu, 740. Edifício Leitão da Cunha. Disciplina de Genética.

Vila Clementino, São Paulo, SP. CEP 04023-900

Telephone number: +55 11 5576 4848.

E-mail: [dgmelo@unifesp.br](mailto:dgmelo@unifesp.br)

**Authors’ ORCID**

Nicoly Stefani Sevalho Carlucci - 0000-0002-6900-7441

Bianca Pereira Favilla - 0000-0002-3988-7708

Beatriz Carvalho Nunes - 0000-0002-1230-1113

Fernanda Teresa de Lima - 0000-0001-9266-9964

Maria Isabel Melaragno - 0000-0002-4344-9698

Rui Fernando Pilotto - 0000-0001-6835-8138

Lucimar Retto da Silva de Avó - 0000-0001-7282-420X

Carla Maria Ramos Germano - 0000-0001-5030-7164

Débora Gusmão Melo - 0000-0001-7005-3544

**Family Sociodemographic Profile Questionnaire**

Regarding your family, please answer the questions below:

What city and state in Brazil do you live in? _____________________________________________________________________________

How many people live in your house? _____________________________________________________________________________

Considering the people who live in your house, how many people are children or teenagers (under 18)?

_____________________________________________________________________________

Considering the people who live in your house, how many people are elderly (over 60 years old)?

_____________________________________________________________________________

Does your family gain any kind of welfare benefits? For example, Family benefit, Organic Law of Social Assistance benefit, Continued benefit, etc.? ( ) Yes ( ) No

What is your family's monthly income?

( ) Up to R$2,090.00

( ) Between R$2,090.01 and R$4,180.00

( ) Between R$4,180.01 and R$10,450.00

( ) Between R$10,450.01 and R$20,900.00

( ) Above R$20,900.01

Does your family usually go to religious services, for example, attending Mass or Evangelical cults?

( ) Yes ( ) No

If affirmative, what religion do you profess?

________________________________________________________________

Does your family have a private health insurance?

( ) Yes ( ) No

In case of a positive answer:

( ) The health insurance covers all members of our family

( ) It is a health insurance linked to the company (work) and only covers the person who works

( ) Only children have health insurance

( ) Only the person with disabilities have health insurance

( ) Only the elderly have health insurance

( ) Other, please describe: _________________________________________________________

Is the treatment of the person with Rett syndrome carried out in the same city that you live in?

( ) Yes ( ) No

About the PARENTS of the person who has Rett syndrome:

MOTHER and FATHER are married; do they live together?

( ) Yes ( ) No

MOTHER and FATHER are related to each other (related as cousins, etc.)?

( ) Yes ( ) No

In an affirmative case, what is the degree of kinship?

___________________________________________________________

Regarding the MOTHER of the person who has Rett syndrome

Age: __________________________

Educational level:

( ) Incomplete secondary education

( ) Incomplete high school

( ) Complete high school, technical, or incomplete higher education

( ) Complete higher education or graduate studies

Work:

( ) Works outside the home full or part-time.

( ) Is currently unemployed.

( ) Does not work outside the home.

Regarding the FATHER of the person who has Rett syndrome

Age: __________________________________

Education level:

( ) Incomplete secondary education

( ) Incomplete high school

( ) Complete high school, technical, or incomplete higher education

( ) Complete higher education or graduate studies

Work:

( ) Works outside the home full or part-time.

( ) Is currently unemployed.

( ) Does not work outside the home.

About the SIBLINGS of the person who has Rett syndrome

( ) The person with Rett syndrome is an only child.

( ) The person with Rett syndrome has siblings.

How many brothers or sisters? ____________________________________________

Do any of the siblings have Rett syndrome?

( ) Yes ( ) No

**Profile of the Person with Rett Syndrome (RTT) questionnaire**

Regarding your child who has RTT, please answer the questions below:

Age: ________________________________________

Gender: ( ) female ( ) male

Was the diagnosis of RTT confirmed by genetic testing?

( ) Yes ( ) No

Was the mutation detected?

( ) Yes ( ) No

If affirmative, which mutation was detected?

( ) MECPP2 ( ) Another gene, please describe: ___________________

Child’s type of school:

( ) She attends regular school.

( ) She attends a special school.

( ) She does not go to school

Education of the person with Rett syndrome:

( ) Less than 8 years.

( ) She is literate (knows how to read and write).

( ) She is illiterate (cannot read and write).

Does your child have seizures?

( ) No

( ) Yes, but they are now controlled without needing to use medications

( ) Yes, the seizures are controlled with medications

( ) Yes, the seizures are not controlled yet, even with medications

Which medication is used for the seizures? ________________________________________________________________________________________________________________________________________________________________

Does the child who has Rett syndrome have any back problems (scoliosis or kyphosis)?

( ) Yes ( ) No ( ) We don’t know

If your answer is positive, did she need surgery to correct it?

( ) Yes ( ) No ( ) We don’t know

Does the person with Rett syndrome have a good relationship with other people of the same age (peers)?

( ) Yes ( ) No

Is the person with Rett syndrome aggressive?

( ) Yes ( ) No

Does the person with Rett syndrome have a difficult day-to-day life?

( ) Yes ( ) No

Does the person with Rett syndrome use any medications because of their behavior?

( ) Yes ( ) No

If so, which one?

________________________________________________________________________________________________________________________________________________________________

Do you think the person with Rett syndrome communicates well?

( ) This is not the case; she is too young for that yet (she is 3 years old or less).

( ) Yes, perfectly. Everyone understands what she says.

( ) More or less. Usually, people in the family or those who live with her understand what she says, but strangers find it difficult to understand.

( ) It's very difficult to understand what she says, but she communicates in a non-verbal way and people in the family or who live with her understand what she means, but strangers don't understand.

( ) She communicates well non-verbally and everyone understands what she means.

( ) No. It is very difficult to understand what she means, whether using words or non-verbal language.

Do you consider that the person with Rett syndrome is autonomous concerning activities of daily living at home? For example, does she shower alone, use the bathroom alone, dress alone, eat alone?

( ) This is not the case; she is too young for that yet (she is 8 years old or younger).

( ) Yes, she is totally autonomous to do this kind of thing.

( ) More or less. She does these things, but she needs supervision.

( ) No, she is unable to do these things alone.

Do you consider your child with Rett syndrome to be autonomous concerning activities of daily living outside the home? For example, she goes to the bakery or runs other errands in the neighborhood alone; if she is of legal age, does she use public transport alone; does she work?

( ) That's not the case; she's too young for that yet (she is 12 years old or younger).

( ) Yes, she is completely autonomous to do this kind of thing.

( ) More or less. She does some of these things, but she needs supervision.

( ) No, she is unable to do these things alone.

Does your child with Rett syndrome know how to handle money? Do you know how to give and receive change?

( ) That is not the case; she is too young for that yet (she is 12 years old or younger).

( ) Yes, she knows how to handle money perfectly.

( ) More or less, sometimes she gets confused.

( ) No, she is incapable of handling money.

**Table S1**. Responses to individual BCFQoLS items among participants (n=70).

| **BCFQoLS domains and questions** | **1**  **Very dissatisfied**  **N (%)** | **2**  **Dissatisfied**  **N (%)** | **3**  **Neither**  **N (%)** | **4**  **Satisfied**  **N (%)** | **5**  **Very satisfied**  **N (%)** |
| --- | --- | --- | --- | --- | --- |
| **Family interaction** | | | | | |
| 1. My family enjoys spending time together | 3  (4.3) | 1  (1.4) | 12  (17.1) | 31  (44.3) | 23  (32.9) |
| 1. My family members talk openly with each other | 3  (4.3) | 6  (8.6) | 17  (24.3) | 31  (44.3) | 13  (18.6) |
| 1. Our family solves problems together | 3  (4.3) | 10  (14.3) | 14  (20.0) | 31  (44.3) | 12  (17.1) |
| 1. My family members support each other to accomplish goals | 3  (4.3) | 6  (8.6) | 14  (20.0) | 32  (45.7) | 15  (21.4) |
| 1. My family members show that they love and care for each other | 3  (4.3) | 3  (4.3) | 3  (4.3) | 32  (45.7) | 29  (41.4) |
| 1. My family is able to handle life's ups and downs | 1  (1.4) | 5  (7.1) | 13  (18.6) | 43  (61.4) | 8  (11.4) |
| **Parenting** | | | | | |
| 1. My family members help the children learn to be independent | 3  (4.3) | 4  (5.7) | 13  (18.6) | 37  (52.9) | 13  (18.6) |
| 1. My family members help the children with schoolwork and activities | 7  (10.0) | 11  (15.7) | 14  (20.0) | 27  (38.6) | 11  (15.7) |
| 1. My family members teach the children how to get along with others | 3  (4.3) | 6  (8.6) | 2  (2.9) | 36  (51.4) | 23  (32.9) |
| 1. Adults in my family teach the children to make good decisions | 0  (0) | 6  (8.6) | 18  (25.7) | 33  (47.1) | 13  (18.6) |
| 1. Adults in my family know other people in the children's lives (i.e. friends, teachers) | 1  (1.4) | 3  (4.3) | 10  (14.3) | 42  (60.0) | 14  (20.0) |
| 1. Adults in my family have time to take care of the individual needs of every child | 2  (2.9) | 9  (12.9) | 12  (17.1) | 35  (50.0) | 12  (17.1) |
| **Emotional well-being** | | | | | |
| 1. My family has the support we need to relieve stress | 8  (11.4) | 10  (14.3) | 18  (25.7) | 25  (35.7) | 9  (12.9) |
| 1. My family members have friends or others who provide support | 9  (12.9) | 15  (21.4) | 17  (24.3) | 20  (28.6) | 9  (12.9) |
| 1. My family members have some time to pursue their own interests | 4  (5.7) | 11  (15.7) | 21  (30) | 26  (37.1) | 8  (11.4) |
| 1. My family has outside help available to us to take care of special needs of all family members | 9  (12.9) | 16  (22.9) | 22  (31.4) | 15  (21.4) | 8  (11.4) |
| **Physical/material well-being** | | | | | |
| 1. My family members have transportation to get to the places they need to be | 5  (7.1) | 6  (8.6) | 5  (7.1) | 25  (35.7) | 29  (41.4) |
| 1. My family gets dental care when needed | 6  (8.6) | 8  (11.4) | 10  (14.3) | 26  (37.1) | 20  (28.6) |
| 1. My family gets medical care when needed | 2  (2.9) | 7  (10.0) | 10  (14.3) | 33  (47.1) | 18  (25.7) |
| 1. My family has a way to take care of our expenses | 3  (4.3) | 7  (10.0) | 18  (25.7) | 29  (41.4) | 13  (18.6) |
| 1. My family feels safe at home, work, school, and in our neighborhood | 3  (4.3) | 7  (10.0) | 12  (17.1) | 32  (45.7) | 16  (22.9) |
| **Disability-related support** | | | | | |
| 1. My family member with special needs has support to make progress at school or workplace | 6  (8.6) | 8  (11.4) | 8  (11.4) | 33  (47.1) | 15  (21.4) |
| 1. My family member with special needs has support to make progress at home | 5  (7.1) | 2  (2.9) | 10  (14.3) | 34  (48.6) | 19  (27.1) |
| 1. My family member with special needs has support to make friends | 6  (8.6) | 3  (4.3) | 10  (14.3) | 35  (50.0) | 16  (22.9) |
| 1. My family has a good relationship with the service providers who work with our family member with a disability | 0  (0) | 1  (1.4) | 14  (20.0) | 36  (51.4) | 19  (27.1) |

**Table S2**. Responses to individual items in the physical and emotional dimensions of the PedsQL 4.0 among participants (n=70).

| **PedsQL 4.0 dimensions and questions** | **100**  **Never**  **N (%)** | **75**  **Almost Never**  **N (%)** | **50**  **Sometimes**  **N (%)** | **25**  **Often**  **N (%)** | **0**  **Almost Always**  **N (%)** |
| --- | --- | --- | --- | --- | --- |
| **Physical functioning (problems with...)** | | | | | |
| 1. Walking more than one block | 14  (20.0) | 5  (7.1) | 13  (18.6) | 8  (11.4) | 30  (42.9) |
| 1. Running | 19  (27.1) | 3  (4.3) | 4  (5.7) | 6  (8.6) | 38  (54.3) |
| 1. Participating in sports activity or exercise | 9  (12.9) | 5  (7.1) | 9  (12.9) | 7  (10.0) | 40  (57.1) |
| 1. Lifting something heavy | 15  (21.4) | 5  (7.1) | 4  (5.7) | 4  (5.7) | 42  (60.0) |
| 1. Taking a bath or shower by him or herself | 21  (30.0) | 1  (1.4) | 0  (0) | 2  (2.9) | 46  (65.7) |
| 1. Doing chores around the house | 23  (32.9) | 2  (2.9) | 0  (0) | 3  (4.3) | 42  (60.0) |
| 1. Having hurts or aches | 7  (10.0) | 15  (21.4) | 26  (37.1) | 13  (18.6) | 9  (12.9) |
| 1. Low energy level | 12  (17.1) | 21  (30.0) | 26  (37.1) | 6  (8.6) | 5  (7.1) |
| **Emotional functioning (problems with…)** | | | | | |
| 1. Feeling afraid or scared | 7  (10.0) | 34.3  (24) | 31  (44.3) | 7  (10.0) | 1  (1.4) |
| 1. Feeling sad or blue | 7  (10.0) | 31  (44.3) | 27  (38.6) | 4  (5.7) | 1  (1.4) |
| 1. Feeling angry | 9  (12.9) | 26  (37.1) | 25  (35.7) | 5  (7.1) | 5  (7.1) |
| 1. Trouble sleeping | 5  (7.1) | 22  (31.4) | 23  (32.9) | 13  (18.6) | 7  (10.0) |
| 1. Worrying about what will happen to him or her | 42  (60.0) | 17  (24.3) | 5  (7.1) | 4  (5.7) | 2  (2.9) |

**Table S3.** Mean scores for each item of the BCFQoLS and the physical and emotional dimensions of the PedsQL among families of children with RTT (n=70).

| **Domains and questions** | **Mean** | **Standard deviation** |
| --- | --- | --- |
| **BCFQoLS - Family interaction** | **3.80** | **0.78** |
| 1. My family enjoys spending time together | 4.00 | 0.98 |
| 1. My family members talk openly with each other | 3.64 | 1.02 |
| 1. Our family solves problems together | 3.56 | 1.07 |
| 1. My family members support each other to accomplish goals | 3.71 | 1.04 |
| 1. My family members show that they love and care for each other | 4.16 | 1.00 |
| 1. My family is able to handle life's ups and downs | 3.74 | 0.81 |
| **BCFQoLS - Parenting** | **3.74** | **0.67** |
| 1. My family members help the children learn to be independent | 3.76 | 0.97 |
| 1. My family members help the children with schoolwork and activities | 3.34 | 1.21 |
| 1. My family members teach the children how to get along with others | 4.00 | 1.05 |
| 1. Adults in my family teach the children to make good decisions | 3.76 | 0.86 |
| 1. Adults in my family know other people in the children's lives (i.e. friends, teachers) | 3.93 | 0.80 |
| 1. Adults in my family have time to take care of the individual needs of every child | 3.66 | 1.01 |
| **BCFQoLS - Emotional well-being** | **3.16** | **0.96** |
| 1. My family has the support we need to relieve stress | 3.24 | 1.20 |
| 1. My family members have friends or others who provide support | 3.07 | 1.24 |
| 1. My family members have some time to pursue their own interests | 3.33 | 1.06 |
| 1. My family has outside help available to us to take care of special needs of all family members | 2.96 | 1.20 |
| **BCFQoLS - Physical/material well-being** | **3.75** | **0.86** |
| 1. My family members have transportation to get to the places they need to be | 3.96 | 1.22 |
| 1. My family gets dental care when needed | 3.66 | 1.25 |
| 1. My family gets medical care when needed | 3.83 | 1.02 |
| 1. My family has a way to take care of our expenses | 3.60 | 1.04 |
| 1. My family feels safe at home, work, school, and in our neighborhood | 3.73 | 1.06 |
| **BCFQoLS - Disability-related support** | **3.81** | **0.85** |
| 1. My family member with special needs has support to make progress at school or workplace | 3.61 | 1.20 |
| 1. My family member with special needs has support to make progress at home | 3.86 | 1.08 |
| 1. My family member with special needs has support to make friends | 3.74 | 1.13 |
| 1. My family has a good relationship with the service providers who work with our family member with a disability | 4.04 | 0.73 |
| **PedsQL 4.0 – Physical functioning (problems with…)** | **38.6** | **28.2** |
| 1. Walking more than one block | 37.5 | 39.4 |
| 1. Running | 35.4 | 43.9 |
| 1. Participating in sports activity or exercise | 27.1 | 36.8 |
| 1. Lifting something heavy | 31.1 | 42.2 |
| 1. Taking a bath or shower by him or herself | 31.8 | 46.0 |
| 1. Doing chores around the house | 36.1 | 46.9 |
| 1. Having hurts or aches | 49.3 | 28.9 |
| 1. Low energy level | 60.4 | 27.4 |
| **PedsQL 4.0 – Emotional functioning (problems with…)** | **63.9** | **14.5** |
| 1. Feeling afraid or scared | 60.4 | 21.5 |
| 1. Feeling sad or blue | 63.9 | 20.3 |
| 1. Feeling angry | 60.4 | 26.1 |
| 1. Trouble sleeping | 51.8 | 27.4 |
| 1. Worrying about what will happen to him or her | 83.2 | 25.8 |

**Table S4**. Bonferroni post hoc comparisons between the domains of the Beach Center Family Quality of Life Scale.

| **Domains** | | **Mean difference** | **95% CI for mean difference** | | **SE** | **t** | **p-value** |
| --- | --- | --- | --- | --- | --- | --- | --- |
|  |  |  | **Lower** | **Upper** |  |  |  |
| Family interaction | Parenting | 0.062 | -0.190 | 0.313 | 0.058 | 1.059 | 1.000 |
|  | Emotional well-being | 0.645 | 0.394 | 0.896 | 0.087 | 7.397 | <0.001*** |
|  | Physical/material well-being | 0.048 | -0.203 | 0.299 | 0.090 | 0.530 | 1.000 |
|  | Disability-related support | -0.012 | -0.263 | 0.239 | 0.101 | -0.120 | 1.000 |
| Parenting | Emotional well-being | 0.583 | 0.332 | 0.835 | 0.076 | 7.635 | <0.001*** |
|  | Physical/material well-being | -0.014 | -0.265 | 0.237 | 0.091 | -0.153 | 1.000 |
|  | Disability-related support | -0.074 | -0.325 | 0.177 | 0.084 | -0.882 | 1.000 |
| Emotional well-being | Physical/material well-being | -0.597 | -0.848 | -0.346 | 0.099 | -6.034 | <0.001*** |
|  | Disability-related support | -0.657 | -0.908 | -0.406 | 0.104 | -6.302 | <0.001*** |
| Physical/material well-being | Disability-related support | -0.060 | -0.311 | 0.191 | 0.089 | -0.677 | 1.000 |

***p<0.001.

**Table S5.** AIC/BIC values from regression models after sequential removal of variables with higher p-values.

| **Predictor** | **𝝱 - Initial model** | **SE** | **t** | **p** | **AIC when predictor is removed** | **BIC when predictor is removed** | **R2** | **R2-adjusted** | **Significant predictor in model** |
| --- | --- | --- | --- | --- | --- | --- | --- | --- | --- |
| Social Benefit | 0.037 | 0.222 | 0.165 | 0.869 | 130.050 | 154.783 | 0.387 | 0.296 | None |
| Father has completed higher education | -0.089 | 0.173 | -0.514 | 0.609 | 128.402 | 150.887 | 0.384 | 0.304 | None |
| Mother has completed higher education | 0.129 | 0.222 | 0.581 | 0.564 | 126.612 | 146.849 | 0.383 | 0.313 | Aggressiveness |
| Private health insurance | 0.183 | 0.181 | 1.009 | 0.317 | 126.270 | 144.258 | 0.368 | 0.308 | Family income, aggressiveness |
| Mother works outside the home | 0.191 | 0.178 | 1.074 | 0.287 | 126.413 | 142.153 | 0.348 | 0.297 | Family income, aggressiveness |
| Father works outside the home | 0.221 | 0.193 | 1.147 | 0.256 | 126.247 | 139.738 | 0.331 | 0.290 | Family income, aggressiveness |
| Age of the person with RTT | -0.016 | 0.013 | -1.228 | 0.224 | 127.604 | 138.846 | 0.298 | 0.266 | Attending regular school, family income, aggressiveness |
| Person with RTT attends regular school | 0.230 | 0.151 | 1.525 | 0.133 | 131.208 | 140.202 | 0.239 | 0.217 | Family income, aggressiveness |
| Monthly family income above four official Brazilian minimum wages | 0.290 | 0.185 | 1.565 | 0.123 | 139.810 | 146.556 | 0.115 | 0.102 | Aggressiveness |
| Aggressiveness | -0.451 | 0.227 | -1.982 | 0.052 | NA | NA | NA | NA | NA |

NA: Not applicable

1. Nicoly Stefani Sevalho Carlucci, Bianca Pereira Favilla, Beatriz Carvalho Nunes, and Fernanda Teresa de Lima should be considered joint first authors. [↑](#footnote-ref-1)
